# Hyperbaric Oxygen Therapy in Experimental Autoimmune Myocarditis: Insights from Preclinical Models to Translational Perspectives

**DOI:** 10.3390/pathophysiology33010018

**Published:** 2026-02-14

**Authors:** Bozidar Pindovic, Vladimir Zivkovic, Radisa Pavlovic, Djurdjina Petrovic, Maja Muric, Ivan Srejovic, Dmitry Kolesov, Marina Kolotilova, Sergey Bolevich, Zarko Finderle, Vladimir Jakovljevic, Aleksandra Stojanovic

**Affiliations:** 1 Department of Pharmacy, Faculty of Medical Sciences, University of Kragujevac, 34000 Kragujevac, Serbia; pindovic.bozidar@gmail.com (B.P.); vranicaleksandra90@gmail.com (A.S.); 2 Center of Excellence for the Study of Redox Balance in Cardiovascular and Metabolic Disorders, Faculty of Medical Sciences, University of Kragujevac, 34000 Kragujevac, Serbia; 3 Department of Physiology, Faculty of Medical Sciences, University of Kragujevac, 34000 Kragujevac, Serbia; 4 Department of Pharmacology, I.M. Sechenov First Moscow State Medical University, 119435 Moscow, Russia; 5 Department of Physical Culture, I.M. Sechenov First Moscow State Medical University, 119991 Moscow, Russia; 6 Department of Pathological Physiology, I.M. Sechenov First Moscow State Medical University, 119991 Moscow, Russia; 7 Department of Human Pathology, I.M. Sechenov First Moscow State Medical University, 119991 Moscow, Russia; 8 Institute of Physiology, Faculty of Medicine, University of Ljubljana, 1000 Ljubljana, Slovenia

**Keywords:** experimental autoimmune myocarditis, hyperbaric oxygen therapy, immune modulation, oxidative stress, animal studies, cardiac inflammation

## Abstract

Myocarditis is still a major global health issue that frequently manifests due to oxidative stress, immune-mediated myocardial damage, and unpredictable clinical progression. Experiments with autoimmune myocarditis (EAM) models have shown different ways that T-cell subsets, proinflammatory cytokines, macrophage polarization, and mitochondrial dysfunction are all connected and play a part in both acute inflammation and chronic remodeling of the heart. As a possible multimodal intervention that could affect several of these disease-causing pathways, hyperbaric oxygen therapy (HBOT) has become popular. This therapy delivers 100% oxygen to different tissues at higher atmospheric pressures. Early research shows that HBOT improves the delivery of oxygen to the inflamed myocardium, suppress the activation of NF-κB and NLRP3 inflammasomes, lowers oxidative stress, protects mitochondrial function, and boosts immune-regulatory T-cell responses. Despite these potentially promising findings, there are still a number of important translational obstacles to overcome, such as inconsistent protocols, a lack of long-term outcome data, insufficient mechanistic profiling, and doubts about the best protocol length and patient selection. To assess safety and effectiveness in human myocarditis, future studies should aim to integrate multi-omics analyses, HBOT regimens that are already standardized, sophisticated imaging, and carefully planned early-phase clinical trials. Overall, the currently available evidence supports HBOT as a biologically plausible and potentially valuable adjunct therapy for autoimmune myocarditis, expressing the need for further mechanistic and clinical investigation.

## 1. Introduction

Whether caused by an infectious trigger or an immune-mediated process, myocarditis continues to be a significant global health issue. Even though diagnostic tools have improved, a lot of cases are still missed, in part because early changes in the myocardium that cause inflammation can be clinically silent [1]. When the disease becomes clinically evident, the consequences are often severe. Approximately 1.32 million people worldwide developed myocarditis, with an age-standardized incidence of roughly 16 cases per 100,000, according to the 2021 Global Burden of Disease analysis. The highest number of new cases were reported in high-income regions of North America and Asia Pacific, while Central Asia displayed a noticeable upward trend, indicating underlying environmental or regional factors that need further investigation [1,2]. Athletes are especially affected by the disease: between 5 and 20 percent of sudden cardiac deaths in young athletes are caused by myocarditis [2].

Even with improved cardiac Magnetic Resonance Imaging (MRI) protocols and more accessible endomyocardial biopsy, fulminant myocarditis continues to carry a high mortality rate. Roughly one fifth of affected patients either die or require transplantation within a year of diagnosis [3]. The clinical course is unpredictable. Although viral infections continue to be the most frequent cause of myocarditis, autoimmune disorders, drug reactions, and idiopathic causes are also significant contributors. Many patients go through an early phase that often goes unnoticed, during which inflammation and changes in the heart’s structure, such as mitochondrial dysfunction and extracellular matrix remodeling, are already happening [4]. This gap between what’s happening in the heart and what doctors can detect shows why we need experimental models that can pinpoint cause-and-effect pathways and test treatments in a controlled way [4]. The experimental autoimmune myocarditis (EAM) model has become one of the best tools for studying how the immune system can damage the heart. In EAM, susceptible rodents are usually immunized with heart muscle proteins or specific peptides mixed with an adjuvant [5]. Numerous important aspects of human myocarditis are replicated in this model, such as the infiltration of dense immune cells (particularly Cluster of Differentiation (CD4+) T cells and macrophages), the death of heart muscle cells, and the subsequent deposition of collagen. Researchers can observe the progression of the disease from acute inflammation to chronic dilated cardiomyopathy because these changes occur in a predictable timeframe [3,4,5]. Because of this clear timeframe, the model has been very useful for studying cytokines, antigen presentation, and the interactions between the innate and adaptive immune systems in cardiac tissue [4]. However, the EAM model has important constraints. The requirement for strong adjuvants, such as complete Freund’s adjuvant, can introduce an exaggerated innate immune response not representative of human disease [5]. Genetic restrictions tied to Major Histocompatibility Complex (MHC) haplotypes mean that certain findings do not readily generalize across strains or species. In particular, the model does not account for viral replication, which is a critical initiating factor in numerous patient populations. These limitations do not diminish EAM’s value but do highlight the need to interpret its results within a translational framework that accounts for what the model can, and cannot, reproduce [3,4,5].

Hyperbaric oxygen therapy (HBOT) offers a mechanistically distinct intervention worth examining in the context of myocarditis. During HBOT, individuals inhale pure oxygen at pressures two to three times greater than atmospheric levels [6]. This short exposure drives arterial oxygen tension to extremely high values, reaching roughly 1400 mmHg at 2.0 atmospheres absolute and exceeding 2200 mmHg at 3.0 atmospheres [7]. Under these conditions, oxygen dissolves directly into plasma at concentrations that can temporarily sustain tissue metabolism independently of hemoglobin-bound oxygen [6,7]. The physiological effects extend beyond oxygen delivery. HBOT has been shown to affect several important pathways in the body. It can boost antioxidant enzymes like superoxide dismutase and catalase, lower pro-inflammatory molecules such as interleukin-1β (IL-1β) and tumor necrosis factor-α (TNF-α), and help mobilize CD34^+^ progenitor cells that are involved in tissue repair [8]. These effects are relevant to myocarditis, where oxidative stress, harmful immune signaling, and poor healing all contribute to heart damage [8].

This review has four main goals. Our first goal was to review the state of knowledge regarding immune-mediated cardiac injury, specifically highlighting the roles played by oxidative stress and mitochondrial dysfunction. The second objective is to provide a comprehensive overview of the results of preclinical studies on HBOT in EAM, which include its impact on molecular pathways, tissue changes, and heart function. Third, to consider how realistic it might be to use HBOT safely and effectively alongside current treatments for myocarditis in human populations. And finally, to point out important areas for future research, such as using advanced molecular profiling, imaging biomarkers, and early clinical trials. By combining insights from immunology, hyperbaric medicine, and heart research, this review aims to provide a clear, evidence-based foundation for exploring HBOT as a treatment for autoimmune and inflammatory heart diseases.

## 2. The Pathophysiology of Autoimmune Myocarditis

Autoimmune myocarditis happens when the immune system gets stuck in a cycle of activation, releasing inflammatory signals and damaging the mitochondria in heart cells. Over time, this leads to scarring and changes in the heart’s structure. Different triggers like viral infections, cancer immunotherapy, or problems with immune regulation can start this process, but the disease usually follows a predictable pattern [9]. It often begins when hidden heart proteins, like α-myosin peptides, are exposed and mistakenly seen as threats by the immune system. Dendritic cells present these proteins to naive T cells, which then move to the heart and create inflammation [10]. At the same time, cytotoxic T cells recognize the same proteins on heart muscle cells and release substances that cause cell death. This cell death releases danger signals, which further stimulate the immune system and worsen the inflammation [10,11,12].

A key step in this process involves Th17s cell, which depend on the presence of the inflammatory molecule interleukin-6 (IL-6) to develop. Blocking IL-6 or removing it genetically in mice prevents myocarditis from developing, while restoring IL-6 brings the disease back. Th17 cells enter the heart and release interleukin-17A (IL-17A), which attracts neutrophils and triggers complement activation, two events that lead to long-term damage and weakening of the heart [11,12]. Other types of T cells release inflammatory molecules like interferon-γ (IFN-γ) and TNF-α, which further compromise cardiac function [12].

Macrophages are another major type of immune cell involved. In the early stages, inflammatory macrophages dominate and release nitric oxide, inflammasome components, and other damaging molecules [12]. If these cells are pushed too far into this state, heart damage gets worse. As healing begins, macrophages shift to a more reparative form that promotes scar formation [13]. However, too much activity in this phase can lead to excessive scarring and stiffness of the heart [12,13].

The activity of different cytokines in autoimmune myocarditis follows a pretty clear timeline. IL-1β and IL-6 appear early and help activate Th17 cells and recruit neutrophils. Research on animals demonstrates that inhibiting IL-1 lowers inflammation and improves heart function [14]. Approximately two to three weeks later, TNF-α and IFN-γ levels rise, keeping immune cells active and causing more damage. If this stage isn’t controlled, the heart starts remodeling in ways that can lead to chronic consequences. Eventually, anti-inflammatory signals like interleukin-10 (IL-10) and transforming growth factor-β (TGF-β) increase, promoting regulatory T cells and helping form scar tissue. While this helps end the acute inflammation, it may also result in long-term cardiac dysfunction [11,12,13,14].

A lot of the damage from autoimmune myocarditis happens in the mitochondria, the cell’s energy factories. Enzymes like nicotinamide adenine dinucleotide phosphate (NADPH) oxidase and disrupted energy production in mitochondria lead to a surge in reactive oxygen species (ROS) [15]. These molecules damage cell membranes, drain energy, and cause mitochondria to break down further. Results from a recent study show that blocking both this oxidative damage and a key mitochondrial stress pathway can protect the heart [16]. Damaged mitochondria also send out signals that further activate immune cells, especially macrophages. In animal studies, higher mitochondrial oxidative stress is associated with impaired heart function, while antioxidants like melatonin and myricetin can reduce damage and promote cardiac recovery [15,16].

Overall, autoimmune myocarditis is a complex process driven by specific immune cells, inflammatory cytokines, and mitochondrial damage. Each of these elements reinforces the others, creating a loop of ongoing injury. Intervening at any point either by blocking harmful cytokines, boosting regulatory T cells, shifting macrophages to a healing state, or protecting mitochondria, has been shown to reduce the effects of this disease in animal studies [17]. This is why therapies that work on multiple parts of the disease process, such as HBOT, look promising. HBOT has been shown to reduce key inflammatory pathways, restore immune balance, and strengthen antioxidant defenses, making it an attractive candidate for further research of this condition [18,19].

### Fibrotic Remodeling in Autoimmune Myocarditis and Potential Modulation by HBOT

The primary feature of chronic myocarditis is myocardial fibrosis, which results from numerous intricate signaling cascades along the TGF-β/SMAD axis. When TGF-β binds to its receptors, it phosphorylates SMAD2/3. This then forms a complex with SMAD4, which then translocate to the nucleus to regulate gene expression. This lets fibroblasts start working and extracellular matrix to form. Endothelial-to-mesenchymal transition (EndMT) further causes the expansion of fibroblasts and progression of fibrosis [20,21]. There are limited data regarding interconnections of myocarditis, HBOT and fibrosis, but in other organ systems HBOT could influence TGF-β/SMAD signaling. For example, HBOT decreased the expression of TGF-β and SMAD4 in lipopolysaccharide-induced endotoxemia models, resulting in a downregulation of α-smooth muscle actin. This suggests that HBOT may have a potential anti-fibrotic effect by modulating the profibrotic pathway [22].

Additionally, HBOT was found to reduce fibrosis and remodeling in diabetic cardiac models, likely through the inhibition of pro-inflammatory cytokines and the modulation of matrix metalloproteinases (MMPs) and TGF-β expression [23]. Overall, this points to a plausible role for HBOT in reducing fibrotic remodeling by regulating the immune, oxidative, and profibrotic pathways. However, further research on myocarditis is required to validate these effects. Since remodeling typically predominates during the subacute/chronic phase of myocarditis, HBOT’s potential antifibrotic effects are likely to be depend largely on the disease stage and timing of treatment.

## 3. Mechanisms of Hyperbaric Oxygenation in Inflammation and Tissue Repair

As mentioned before, HBOT boosts the oxygen level in the blood to around 2000 mmHg at typical treatment pressures. By getting to inflammatory or undersupplied parts of the heart, this additional oxygen can help heart cells get the energy they require, activate protective mechanisms like the production of antioxidant enzymes, and reduce hyperactive immune responses [24]. This high oxygen level allows oxygen to reach areas of the heart that are inflamed, swollen, or poorly supplied with blood, which is often present in myocarditis [8]. In this way, HBOT helps overcome local oxygen shortages and protects heart tissue from damage [25].

HBOT also affects important inflammatory pathways. It prevents the production of pro-inflammatory substances like TNF-α, IL-1β, IL-6, and Intercellular Adhesion Molecule-1 (ICAM-1). Additionally, it has been demonstrated that HBOT inhibits the activation of the NOD-, LRR-, and pyrin domain-containing protein 3 (NLRP3) inflammasome, which is a major inducer of inflammation in heart injury [26]. Looking beyond just reducing inflammation, HBOT supports tissue repair and also helps stabilize hypoxia-inducible factor 1α (HIF-1α), a protein that encourages new blood vessels to grow. This supports the recruitment of stem cells that repair damaged blood vessels and promote healing in the heart [27]. In addition, HBOT protects mitochondria by keeping key enzymes active, stopping energy loss, and stopping pathways that lead to cell death. This protective effect is particularly significant in myocarditis because mitochondrial damage is a major cause of both immune system activation and heart cell death [28]. It lowers the number of harmful Th17 immune cells and in-creases the presence of regulatory T cells, which helps in keeping inflammation in check. This shift has been observed in several models of autoimmune disease [29]. The benefits of HBOT, including enhanced oxygen delivery, decreased oxidative stress, decreased inflammation, improved blood vessel repair, mitochondrial protection, and immune system rebalancing, collectively offer a compelling and scientifically supported argument for investigating it as either a possible treatment or adjunctive treatment for autoimmune myocarditis. Because many of these mechanisms depend on the magnitude of oxygen tension achieved during treatment, the approximate arterial oxygen levels produced at different hyperbaric pressures are summarized in Table 1.

Collectively, these preclinical findings support HBOT as a potent multi-modal modulator of immune and oxidative pathways in EAM, providing a compelling rationale for further mechanistic and translational studies to optimize therapeutic regimens and explore applicability to human autoimmune myocarditis.

Multiple studies have investigated the effects of different HBOT pressures and durations across a variety of diseases, demonstrating that both therapeutic efficacy and potential toxicity are dose- and pressure-dependent. Experimental data show that while exposures to 2.0–2.5 ATA generally maintain ROS within physiological ranges, pressures around 3.0 ATA can produce higher ROS levels that can saturate antioxidant systems and lead to tissue damage in sensitive organs such as the lungs and central nervous system [33]. Acute central nervous system oxygen toxicity has been documented even at pressures less than 2.0 ATA with high cumulative exposure, showing that total oxygen dose rather than only pressure can cause risk of toxicity [34]. Repeated HBOT sessions at pressures commonly used in clinical practice in the range from 2.0–2.5 ATA seem to improve mitochondrial activity and antioxidant defenses, decreasing ROS and improving mitochondrial function over time, probably via increased expression of antioxidant enzymes and mitochondrial biogenesis pathways [33]. Comparisons of 1.4 ATA and 2.5 ATA exposures in humans show a similar acute increase in ROS and antioxidant reactions, with inflammatory markers following the changes to oxygen dose, showing that a biological response depending on the oxygen dose is still not very well documented for pressures above 2.5 ATA [35]. When pressures go above therapeutic ranges such as 2.5–3.0 ATA and are prolonged, there is evidence of greater oxidative stress and saturation of antioxidants, which most preclinical models link with oxidative damage and potential organ toxicity [36].

### Oxidative Paradox and Redox Dependent Dose Effects of HBOT

HBOT raises the oxygen tension in tissues by increasing the amount of oxygen dissolved in plasma and tissue fluids. This causes systemic and local tissue hyperoxia that is not caused by hemoglobin delivering oxygen [33,34,35,36]. This acute hyperoxic state initially stimulates the generation of reactive nitrogen species (RNS) and ROS by mitochondria and enzymatic processes, resulting in a dose- and time-dependent increase in oxidative burden during the exposure [31,36,37]. This aligns with the hyperoxic–hypoxic paradox and the mitohormesis principle, which states that transient oxidative stress serves as a signaling stimulus that triggers various adaptive cellular responses instead of resulting in long-term harm [27,37,38]. While excessive or prolonged hyperoxia can overwhelm antioxidant defenses which results in oxidative injury, controlled and intermittent HBOT protocols promote signaling pathways sensitive to redox changes that cause cytoprotection and metabolic adaptation [27,36,38]. In particular, the transitory increased production of ROS activates nuclear factor erythroid 2-related factor 2 (Nrf2), a redox sensitive transcription factor that functions as a master regulator of antioxidant and cellular defense mechanisms [31,37,38]. After nuclear stabilization activation, Nrf2 initiates the transcription of multiple antioxidant and detoxifying enzymes, including superoxide dismutase (SOD), catalase (CAT), heme oxygenase-1 (HO-1), glutathione peroxidase (GPx), and other enzymes involved in glutathione synthesis and redox homeostasis [39,40,41]. This coordinated upregulation improves ROS scavenging capacity, restores redox balance, and reduces cumulative oxidative damage to lipids, proteins, DNA, and mitochondrial structures, particularly following repeated or intermittent HBOT exposure [27,36,42].

HBOT mediated ROS production is dependent on different conditions and can be influenced by treatment pressure, duration, frequency, and total number of exposures [38,43,44]. Short-term HBOT protocols lasting from one to five sessions at higher oxygen tensions can temporarily increase mitochondrial ROS production, reflecting an initial oxidative challenge associated with excess oxygen availability and enhanced electron leakage from the electron transport chain [43,45,46]. This short-term rise in ROS production can temporarily suppress mitochondrial activity and membrane potential as a form of protective adaptive response, to limit further oxidative damage [43,44]. In contrast, repeated or intermittent HBOT regimens induce adaptation with the activation of cytoprotective signaling pathways and transcription factors, including Nrf2 [47,48], HIF1α [38,49], and SIRT1 [38,50]. This is also followed by the upregulation of enzymatic and nonenzymatic antioxidant defenses in the form of SOD, HO-1, and GPx [51,52]. Over time, this coordinated response reduces net oxidative stress, restores mitochondrial function, and improves general cell redox homeostasis [53,54]. This balance between prooxidant and antioxidant effects offers an explanation for heterogeneous findings reported in the HBOT literature, where some studies demonstrate increased oxidative stress following acute exposure [45,46] while others report antioxidant, antiapoptotic, and metabolic benefits after prolonged or intermittent treatment [53,54]. An explanation for the inconsistent results found in the HBOT literature is provided by this balance between prooxidant and antioxidant effects. While some studies show increased oxidative stress after acute exposure [45,46], others report antioxidant, antiapoptotic, and metabolic benefits after prolonged or intermittent treatment [53,54]. Therefore, when using HBOT in areas with immune-mediated oxidative injury and mitochondrial dysfunction, dose, timing, and treatment protocol optimization are required [44,55]. In myocarditis, where ROS production, inflammatory immune cell infiltration, nuclear factor kappa-light-chain-enhancer of activated B cells (NF-κB) activity [43,56], and mitochondrial damage together contribute to cardiomyocyte injury and disease progression, the modulating effects of HBOT could prove relevant as a form of treatment [57,58,59,60,61]. Paramount care in choosing HBOT protocols is necessary to make maximum use of the adaptive antioxidant and immunomodulatory benefits while at the same time avoiding exacerbation of oxidative stress in the inflamed myocardium [59,60,61,62,63]. These observations show that HBOT cannot be considered intrinsically antioxidant and may be contraindicated in settings of uncontrolled inflammation or advanced mitochondrial dysfunction, particularly when high pressures or prolonged exposure are used.

## 4. Hyperbaric Oxygen Therapy in Experimental Autoimmune Myocarditis: Preclinical Evidence

**Figure 1:**
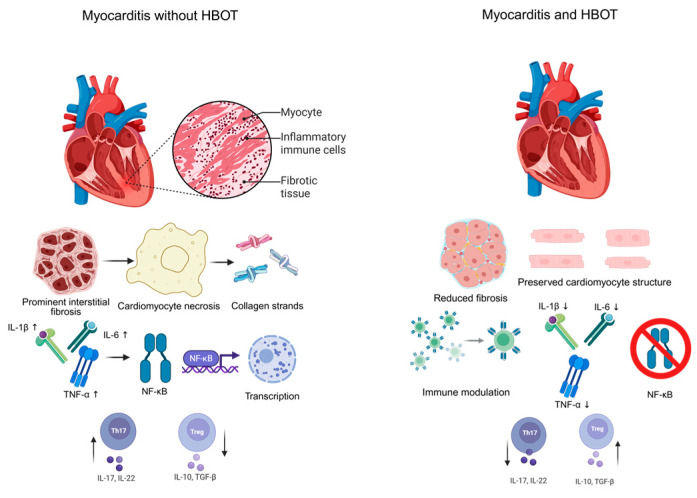
Illustrative scheme of potential HBOT effects in experimental autoimmune myocarditis. ↑—increase; ↓—decrease.

Many EAM models, such as the Galectin-3 (Gal-3)–deficient C57BL/6J mice immunized with MyHCα_334–352_ peptide as employed by Milinčić et al., provide a genetically susceptible platform to study myocarditis characterized by severe mononuclear cell infiltration, necrosis, and pronounced fibrosis due to Gal-3 deficiency [64]. While direct EAM + HBOT evidence in non-Gal-3 deficient mouse lines is limited, more traditional EAM induction protocols in Lewis or Balb/c rats immunized with cardiac myosin peptides are commonly used to investigate cytokine signaling and fibrotic processes [65]. HBOT regimens in these models typically involve administration of 100% oxygen at 2.5 ATA for approximately 90 min per session, delivered daily from days 7 to 21 post immunization during the therapeutic phase, aligning with standard cardiac HBOT protocols in rodent studies [64]. Functional outcomes reported by Milinčić et al. [64] include significantly improved fractional shortening, reduced left ventricular internal diameters at diastole and systole, decreased myocardial remodeling, necrosis, and inflammatory infiltration upon histological examination, alongside improved survival or delayed heart failure progression [64]. Histologically, HBOT-treated hearts show decreased mononuclear infiltration and lower myocarditis scores, preservation of cardiomyocytes with reduced degeneration and necrosis, and significant attenuation of fibrosis compared to untreated EAM animals [64,65]. Although direct cytokine quantification post-HBOT in EAM models remains limited, data from EAM rats treated with anti-inflammatory agents demonstrate downregulation of key cytokines IL-1β, IL-6, and TNF-α via NF-κB pathway inhibition, mechanisms likely shared by HBOT [65]. In addition, studies in experimental autoimmune encephalomyelitis (EAE) show that HBOT can reduce proinflammatory Th17 cells and increase regulatory T cells (Tregs), suggesting that it might have similar immune-balancing effects in myocarditis [29]. These combined immunomodulatory and tissue protective actions of HBOT are summarized in Figure 1.

While these results are encouraging, there are still important limitations. Most studies use very specific mouse strains, like Gal-3 deficient mice, and have small group sizes (fewer than 15 animals), which makes it hard to know if the findings are reliable or repeatable [66]. Many experiments also end at day 21 after immunization, so we don’t yet know how HBOT affects long-term heart changes, arrhythmias, or chronic forms of the disease [67]. Furthermore, no systematic studies have been conducted to determine the optimal HBOT pressure, session length, or total treatment time. This lack of data complicates the comparison of results across studies [68]. Given the limited number of studies directly evaluating HBOT in experimental autoimmune myocarditis, additional evidence from related cardiovascular and inflammatory animal models is discussed to provide mechanistic context rather than direct therapeutic validation. A summary table (Table 2) consolidates key parameters from selected animal studies to illustrate mechanistic effects of HBOT on inflammation, oxidative stress, and tissue injury across cardiovascular and systemic inflammatory models.

An important thing to mention is that the studies summarized in this table do not represent direct evidence from experimental autoimmune myocarditis models. Instead, they are included to illustrate shared inflammatory, oxidative, mitochondrial, and microvascular mechanisms relevant to myocardial injury. They should therefore be interpreted as supportive mechanistic context rather than proof of therapeutic efficacy in autoimmune myocarditis.

## 5. Translational Relevance and Clinical Perspectives

The results from animal studies suggest that HBOT could be beneficial for human myocarditis, which remains challenging to treat due to its heterogeneity and the limited treatment options. In humans, myocarditis is often caused by viral infections or autoimmune disorders, and it shares important features with EAM models, including T-cell driven inflammation, dysregulated cytokine signaling, and oxidative stress induced cardiac damage [72,73]. To date, to the best of our ability we found no clinical trials have evaluated hyperbaric oxygen therapy in patients with myocarditis of any etiology, and all clinical evidence discussed below is therefore indirect and hypothesis-generating. HBOT’s ability to modulate immune responses, reduce oxidative damage, and promote tissue repair through angiogenesis and mitochondrial protection presents a compelling rationale for translational exploration [74].

However, significant barriers complicate the direct translation of animal findings to human clinical use. The timing of the intervention is crucial because, unlike animal models, where HBOT treatment usually begins during the acute inflammatory phase, human patients frequently present with varying stages of the disease, making the exact moment as to when to start the therapy more difficult to determine [75]. Furthermore, access to HBOT chambers and practical issues also make it hard to use widely, especially in urgent heart care [76]. Due to the fact that myocarditis can range from self-limited to fulminant disease forms, which may react differently to HBOT, patient selection criteria are still unclear [9]. Even though EAM models resemble autoimmune myocarditis, they are unable to accurately replicate viral forms or the long-term cardiac abnormalities observed in people [9].

To assess the safety and efficacy of HBOT, future clinical trials need to carefully choose the adequate patients to include. Biomarkers such as blood cytokines, T-cell counts, or advanced imaging should be used to track the results of treatment [75].

To truly test HBOT as an additional treatment or substitute for existing immune-suppressive or antiviral therapies, large, multi-center randomized trials would be required; ideally, these trials would include close examinations of cardiac tissue to identify the underlying mechanisms [77].

### Immune Checkpoint Inhibitor-Associated Myocarditis and HBOT

Immune checkpoint inhibitors (ICIs) may have revolutionized oncology treatment, but they have also been labeled as causes of myocarditis characterized by T-cell hyperactivation, metabolic dysregulation, and fulminant inflammation [78,79]. Although no studies directly assess HBOT in ICI myocarditis, different common mechanistic parallels in redox imbalance, mitochondrial stress suggest biologically plausible interactions [80]. HBOT can affect mitochondrial ROS production and inflammatory pathways, and it can potentially influence immune activation while at the same time promoting regulatory pathways [46,48]. However, the risk of exacerbating oxidative injury in the context of cardiac damage caused by ICI remains uncertain given the lack of direct evidence. Additionally, the potential role of HBOT in ICI-associated myocarditis should currently be used as a starting hypothesis that requires dedicated experimental investigation. Given the fulminant nature of ICI-associated myocarditis, inappropriate timing or dosing of HBOT could theoretically exacerbate myocardial injury.

## 6. Risks, Side Effects, and Patient Selection for Hyperbaric Oxygen Therapy

While HBOT is generally safe, like any medical treatment, it carries certain risks and potential side effects. The most common issue is barotrauma, which mainly affects the middle ear. Changes in pressure during treatment can make the ears uncomfortable or even damage the eardrum if not managed carefully. Patients may also experience claustrophobia due to being in tight spaces while confined in the chamber, which can sometimes require sedation or careful psychological support [76]. Less commonly, breathing high pressure oxygen can cause oxygen toxicity, which could lead to seizures or lung irritation. These serious problems are rare but require close monitoring during sessions. Other possible side effects include temporary changes in vision and fatigue [76].

To keep patients safe, screening is important before starting HBOT. Absolute contraindications include untreated pneumothorax, as the pressure changes can worsen this condition. Relative contraindications include certain lung diseases, uncontrolled seizures, or severe congestive heart failure. Medical history, physical examination, and sometimes imaging are used to assess eligibility [81]. Although HBOT is not yet a standard treatment for myocarditis, if it were to be considered as a therapeutic option in the future, patients would need to be closely monitored for possible adverse effects. Since the incidence of complications can increase with the use of higher pressures (>2.0 ATA) or prolonged treatment courses (>10 sessions), we would need to carefully adjust therapy parameters to meet the needs of the patients [82].

## 7. Current Clinical Indications and Outcomes of Hyperbaric Oxygen Therapy

It has already been established that HBOT is a well-known treatment used for many different medical conditions. It is especially effective for treating decompression sickness, which can occur in divers who surface too quickly, and for carbon monoxide poisoning, where it helps to promptly remove the toxic gas from the blood [83]. In addition, HBOT also has applications in managing diabetic foot ulcers, chronic refractory osteomyelitis, and radiation-induced tissue damage [84].

Studies showed that people with diabetic foot ulcers heal faster and have fewer amputations when treated with HBOT [85]. In chronic bone infections, HBOT supports antibiotic therapy and helps resolve infection by improving blood flow and oxygenation in damaged tissues. Radiation injuries, which often cause persistent tissue damage and poor healing, also respond well to HBOT which reduces pain and tissue necrosis [86].

Overall, large patient cohorts treated with HBOT for radiation-induced injuries have shown high rates of symptom improvement or resolution, ranging from approximately 77% to 93% depending on the type of injury. These outcomes are documented in numerous clinical trials and are supported by international guidelines in hyperbaric medicine [87]. The widespread and well-documented efficacy of HBOT in these diverse conditions provides a strong foundation for exploring its use in other diseases, such as autoimmune myocarditis.

HBOT has also been studied in other cardiovascular diseases beyond myocarditis, both in animal models and clinical settings. In animal studies of cardiac arrest, it improved the chances of restoring spontaneous circulation and reduced brain damage after resuscitation [84]. This suggests that HBOT may protect heart and brain tissues by improving oxygen delivery during critical injury.

In models of ischemic heart disease, HBOT has been shown to reduce inflammation and limit tissue damage by enhancing oxygen supply to areas affected by reduced blood flow [25]. It also appears to promote the formation of new blood vessels and stimulate repair mechanisms [88]. Some early clinical studies suggested potential benefits in conditions such as refractory angina and chronic heart failure, though larger trials are needed to confirm these findings [89]. A recent meta-analysis showed that adjunctive HBOT significantly improved left ventricular ejection fraction (LVEF) in patients with coronary artery disease after revascularization compared with controls, indicating potential benefit in myocardial function recovery [90]. Randomized controlled data from post-COVID-19 patients have demonstrated improvements in left ventricular systolic function (global longitudinal strain) following HBOT compared with sham controls. COVID-19 patients underwent a HBOT protocol that consisted of 40 daily sessions over approximately two months, with five sessions per week. Each session lasted 90 min, during which patients in the treatment group breathed in 100% oxygen at a pressure of 2 ATA, with five-minute breaks every 20 min, and compression/decompression at 1.0 m/min. This supports indications that HBOT can increase recovery of subclinical systolic function in post-COVID-19 patients [72]. Altogether, this suggests potential mechanisms by which HBOT could influence myocardial recovery; however, they do not provide evidence of efficacy in myocarditis.

**Figure 2:**
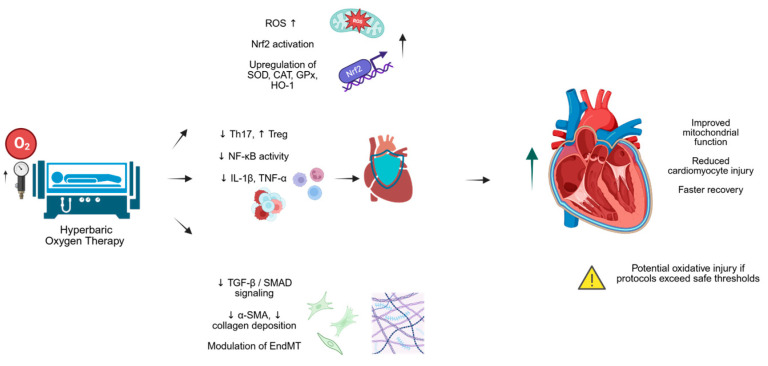
Illustrative summary of potential mechanisms affected by HBOT. ↑—increase; ↓—decrease.

Additionally, HBOT has been found to mitigate vascular dysfunction in animal models of systemic inflammatory conditions, which may translate to benefits in cardiovascular diseases linked to inflammation and oxidative stress [91]. Together, these studies point to a growing interest in applying HBOT to a broader range of cardiovascular disorders, with existing preclinical and initial clinical data supporting its role as an adjunct therapy (Figure 2).

HBOT shows considerable potential as a therapeutic approach for patients with cardiovascular disease, as it targets key mechanisms involved in the development and progression of atherosclerosis, endothelial dysfunction, and inflammation. However, further research is required to support personalized and optimized use of HBOT in cardiovascular care and to clarify its role in the prevention and management of cardiovascular disease [92].

## 8. Hyperbaric Oxygen Therapy and Myocarditis Mechanisms

In myocarditis, the relevance of HBOT lies not in a single pathway but in its capacity to affect immune activation, oxidative signaling, and mitochondrial stress. Several anti-inflammatory effects of HBOT, including attenuation of NF-κB cytokine signaling, are likely secondary to redox-sensitive pathway modulation that was discussed above [93]. At the same time, HBOT promotes the growth and activity of regulatory T cells, which play a crucial role in maintaining the immune system’s balance. These cells help prevent excessive inflammation and tissue damage by counteracting the inflammatory effects of T helper 17 cells. This restoration of balance between different types of immune cells is crucial in controlling myocarditis and preventing its progression [94]. On a cellular level, HBOT also protects mitochondria, the energy centers of heart cells. In myocarditis, mitochondria suffer from oxidative damage, which triggers cell death and worsens heart function. HBOT helps by preserving mitochondrial function, preventing the opening of pores that would otherwise lead to cell death, and reducing the production of harmful ROS. Together, these effects of HBOT directly target the pathways that cause damage in myocarditis, making it a promising therapeutic approach [89]. Different studies in animal models support these findings, showing that HBOT treatment decreases inflammation, reduces oxidative stress, and improves heart function. These results suggest that the mechanisms of HBOT are well-aligned with the complex immunopathology of myocarditis [64,95,96,97].

## 9. Future Directions

Although there have been promising preclinical results about HBOT in EAM, there are still significant knowledge gaps that need to be filled in order to fully realize its translational potential. First, there are issues with generalizability due to the existing body of evidence’s small sample sizes and lack of reproducibility across various animal models, especially outside of Galectin-3 deficient mice [64]. The absence of standardized HBOT treatment protocols further complicates interpretation since variations in pressure, duration, frequency, and timing across studies obscure the identification of optimal therapeutic regimens, pointing to the urgency for dose–response investigations [76]. Moreover, mechanistic insights into HBOT’s immunomodulatory effects remain incomplete. Multi-omics approaches including transcriptomics, proteomics, and metabolomics could elucidate myocardial tissue alterations and identify biomarkers predictive of response [98]. Combining HBOT with already established immunosuppressive treatments is a tenable approach that may target oxidative stress and immune dysregulation at the same time, potentially producing additive or synergistic benefits [29]. Advanced imaging modalities such as cardiac magnetic resonance imaging (MRI) and positron emission tomography (PET) offer powerful noninvasive tools to monitor inflammation, fibrosis, and myocardial remodeling longitudinally, and their integration into HBOT studies could enhance the evaluation of therapeutic efficacy [99]. Additionally, investigating long-term outcomes including cardiac remodeling, arrhythmogenesis, and heart failure progression is crucial, as most current studies focus on acute phase end-points.

## 10. Conclusions

In summary, HBOT exhibits potential as a multitarget therapy that can modify significant immunological and oxidative pathways connected to the pathophysiology of myocarditis; nevertheless, before it can be applied in clinical settings, successful human research is required to get past practical and biological obstacles. By focusing on important inflammatory pathways and reestablishing immunological balance, preclinical data in EAM models shows that HBOT improves cardiac function, reduces inflammation, and reduces remodeling. Despite these promising results, there are still many obstacles to overcome before they can be implemented in clinical settings, such as inconsistent treatment regimens, a lack of functional and causal knowledge, and the requirement for thorough clinical trials. At present, HBOT should be regarded strictly as an experimental and adjunctive strategy in myocarditis, supported by preclinical evidence but lacking direct clinical validation. Future research should focus on standardizing HBOT regimens, exploring combination therapies with established immunosuppressants, and employing advanced imaging and molecular approaches to monitor treatment effects and identify biomarkers. Ultimately, well-designed clinical studies are essential to validate HBOT’s efficacy and safety in human myocarditis, potentially offering a novel therapeutic avenue for this complex and often disabling condition.

## Figures and Tables

**Table 1:** Relationship between pressure in atmospheres absolute (ATA), the breathing gas, and the resulting arterial oxygen tension (PaO_2_) [8,30,31,32].

Approximate Arterial Oxygen Tension at Different Hyperbaric Pressures			
ATA (atm abs)	Breathing Gas	Approximate PaO_2_ (mmHg)	Notes
1.0	Air	100	Normal ambient conditions
1.0	100% O_2_	600	Max dissolved O_2_ at sea level
1.5	100% O_2_	1000	Moderate hyperbaric oxygenation
2.0	100% O_2_	1400	Common therapeutic pressure
2.5	100% O_2_	>2000	Standard HBOT protocol
3.0	100% O_2_	2200–2800	High end clinical HBOT pressure

**Table 2:** The effects of various HBOT protocols tested in animal models, mainly rats, under different pathological conditions.

Effects of Different HBOT Protocols in Animal Studies			
Study	Model/Species	HBOT Regimen	Key Outcome (s)
Oliveira, Mario S et al., 2020 [25]	Rat myocardial ischemia (acute MI model)	2.5 ATA, 60 min post-occlusion	↑ SOD, catalase; ↓ ROS markers; improved survival
Chen, Chunxia et al., 2017 [69]	Rat myocardial model	2.4 ATA, multiple sessions	↓ TNF-α, IL-1β, IL-6; improved mitochondrial integrity
Lee J. Goldstein et al., 2009 [70]	Rat limb ischemia + EPC transplant	2.4 ATA, 3 h/day × 5 days	↑ bone marrow nitric oxide & CD34^+^ EPC → enhanced angiogenesis
Masatomo Y, Mamoru Y, 2000 [71]	Hemorrhagic shock in rats	3.0 ATA, during resuscitation	↓ systemic TNF-α & IL-6 mRNA and serum levels

HBOT—Hyperbaric oxygen therapy, ATA—Atmospheres absolute (unit of pressure), MI—Myocardial infarction, ROS—Reactive oxygen species, SOD—Superoxide dismutase (antioxidant enzyme), TNF-α—Tumor necrosis factor α (pro-inflammatory cytokine), IL-1β, IL-6—Interleukin 1 β/Interleukin 6 (pro-inflammatory cytokines), EPC—Endothelial progenitor cells. ↑—increase; ↓—decrease.

## Data Availability

No new data were created or analyzed in this study. Data sharing is not applicable to this article.

## Abbreviations

The following abbreviations are used in this manuscript:

ATA	Atmospheres Absolute
CD4+	Cluster of Differentiation 4 positive T cells
EAM	Experimental Autoimmune Myocarditis
EAE	Experimental Autoimmune Encephalomyelitis
EPC	Endothelial Progenitor Cells
EndMT	Endothelial-to-Mesenchymal Transition
Gal-3	Galectin-3
HIF-1α	Hypoxia-inducible factor 1-alpha
HBOT	Hyperbaric Oxygen Therapy
HO-1	Heme Oxygenase-1
IFN-γ	Interferon-gamma
ICAM-1	Intercellular Adhesion Molecule-1
ICI	Immune Checkpoint Inhibitors
IL-1β	Interleukin-1 beta
IL-6	Interleukin-6
IL-10	Interleukin-10
IL-17A	Interleukin-17A
MHC	Major Histocompatibility Complex
MI	Myocardial Infarction
MyHCα_334–352_	α-myosin heavy chain peptide (residues 334–352)
MMPs	Matrix Metalloproteinases
MRI	Magnetic Resonance Imaging
NADPH	Nicotinamide Adenine Dinucleotide Phosphate (oxidase)
NLRP3	NOD-, LRR- and pyrin domain-containing protein 3
NF-κB	Nuclear Factor kappa-light-chain-enhancer of activated B cells
Nrf2	Nuclear Factor Erythroid 2–related factor 2
PaO_2_	Arterial Oxygen Tension
PET	Positron Emission Tomography
ROS	Reactive Oxygen Species
RNS	Reactive Nitrogen Species
SMAD	Mothers Against Decapentaplegic Homolog Protein Group
TGF-β	Transforming Growth Factor-beta
Th17	T helper 17 cells
TNF-α	Tumor Necrosis Factor-alpha
Tregs	Regulatory T cells
